# Generation of a Novel MMTV-tTA Transgenic Mouse Strain for the Targeted Expression of Genes in the Embryonic and Postnatal Mammary Gland

**DOI:** 10.1371/journal.pone.0043778

**Published:** 2012-08-31

**Authors:** Kazuhito Sakamoto, Jeffrey W. Schmidt, Kay-Uwe Wagner

**Affiliations:** 1 Eppley Institute for Research in Cancer and Allied Diseases, University of Nebraska Medical Center, Omaha, Nebraska, United States of America; 2 Department of Biochemistry and Molecular Biology, University of Nebraska Medical Center, Omaha, Nebraska, United States of America; 3 Department of Pathology and Microbiology, University of Nebraska Medical Center, Omaha, Nebraska, United States of America; University of Connecticut, United States of America

## Abstract

We have generated a new and improved transgenic mouse strain that permits a temporally controlled expression of transgenes throughout mammary gland development. High expression of the tetracycline-regulatible transactivator (tTA) under control of the mouse mammary tumor virus long terminal repeat (MMTV-LTR) is restricted to mammary epithelial cells and the salivary gland. The novel MMTV-tTA mouse strain induces a sustained transactivation of responder transgenes, which can be swiftly suppressed through administration of doxycycline (Dox). An important characteristic of this strain is its expression in early progenitor cells of mammary gland anlagen beginning at day 13.5 of embryonic development. We show here that the MMTV-tTA can be used in combination with GFP reporter strains to visualize CK8/CK14-dual positive progenitors in newborn females and their derived basal and luminal epithelial cell lineages in adult females. Our observations suggest that the novel MMTV-tTA can be utilized to express exogenous proteins in multipotent mammary progenitors during the earliest stages of mammary gland development to assess their biological significance throughout mammogenesis. Moreover, we demonstrate that the expression of the MMTV-tTA is sustained during mammary gland tumorigenesis in female mice expressing wildtype ErbB2. This makes this strain particular valuable to target the expression of exogenous proteins into developing mammary tumors to assess their significance in biological processes, such as tumor cell growth and survival, metabolism, and metastasis.

## Introduction

Since its adaptation into transgenic mice in the early 1990s [1], the tetracycline (Tet)-controlled expression system has provided a unique way of regulating the activity of transgenes *in vivo*. The Tet-system requires three essential components: 1) a transgene that mediates the expression of the tetracycline-controlled transactivator (tTA) to a particular tissue, 2) a TetO-driven effecter transgene containing the tet-operon linked to a minimal promoter from the human cytomegalovirus immediate early gene 1, and 3) a tetracycline derivative such as doxycycline (Dox). In combination with tissue-specific promoters, the Tet-system now allows for a temporal and spatial regulation of transgenes in a variety of organs.

A first attempt to target the Tet/Dox-repressible transactivator (tTA, Tet-OFF) to the mammary gland was published by Hennighausen and colleagues [2]. The tTA was placed under the control of regulatory elements of a mouse mammary tumor virus long terminal repeat (MMTV-LTR) that was approximately 1.2 kb in size. Although the resulting MMTV-tTA transgene is expressed in the mammary gland, its activity is relatively weak in the mammary epithelium, and there is extensive nonspecific expression of the tTA in numerous other organs, including the skin, salivary gland, thymus, spleen, and pancreas.

A markedly improved tissue-specificity and transactivation of TetO-driven transgenes in the mammary gland was achieved in an MMTV-rtTA transgenic strain that expresses the reverse transactivator (rtTA) under control of a longer MMTV-LTR of approximately 2.4 kb in size [3]. This regulatory element includes a portion of the v-H-ras leader sequence, which has been shown to mediate an enhanced expression of transgenes in the mammary epithelium [4]. Despite strong expression of the rtTA and TetO-driven transgenes to the mammary epithelium, the MMTV-rtTA also shows some expression in the salivary gland and seminal vesicle. It should be noted that this activity, which is commonly referred to as a “leaky expression” of the MMTV-LTR, is actually a necessity as part of the normal life cycle of the MMTV retrovirus and, therefore, essentially unavoidable.

To target the expression of the Tet-controlled transactivator exclusively to the mammary gland, our laboratory developed knockin mice that express the rtTA under control of the endogenous *Whey acidic protein (Wap)* locus. Wap-rtTA transgenics are particularly useful for a strong, ligand-controlled activation of TetO-driven transgenes in alveolar cells during late pregnancy, lactation, and the onset of involution [5], [6]. In contrast to MMTV-rtTA transgenics, the WAP-rtTA-mediated expression is insignificant in nulliparous and in nonpregnant females following postlactational involution. In both, the MMTV-rtTA and the WAP-rtTA, the transactivation of TetO-driven responder genes requires a continuous administration of Dox. While this appears to be no problem when the phenotypic and molecular effects of genes are being examined within a reasonable window of activation, we have observed that a long-term administration of Dox in the food or drinking water for over a year resulted in a poor health of animals that can contribute to experimental variations [7]. In part, this is caused by the sucrose or other high-energy supplements to mask the bitter taste of Dox that can lead to obesity and diabetes. In conclusion, it is technically advantageous and much less expensive to use the tTA to activate TetO-based transgenes in a constitutive manner over long periods in specific tissues. This is particularly important for the study of tumor susceptibility genes with weak oncogenicity.

We describe here the development and characterization of a novel MMTV-tTA transgenic strain that exhibits a strong expression of the Tet/Dox-repressible transactivator (Tet-OFF) in the mammary gland epithelium throughout perinatal and postnatal development. This strain can be utilized to constitutively express TetO-driven responder genes without administration of Dox. The administration of this ligand immediately suppresses the reporter transgene, and its expression is quickly recovered after withdrawal of Dox. A time course analysis shows that the transactivator is stably active in the mammary gland beginning at embryonic stage E14.5, which makes this strain particularly useful to target the expression of transgenes to early mammary gland progenitors in the prenatal mammary gland and beyond. We also confirmed that TetO-driven responder transgenes are active in ErbB2-induced mammary tumors. This strain can, therefore, be applied to express exogenous proteins in mammary tumors to test their biological effects for tumor cell growth, metabolism, and metastatic dissemination of cancer cells.

## Results

### The previously generated MMTV-tTA(NIH) transgenic mouse strain exhibits a wide-spread expression pattern in the FVB genetic background and has limited applicability for activation of responder transgenes in the mammary gland

**Figure 1:**
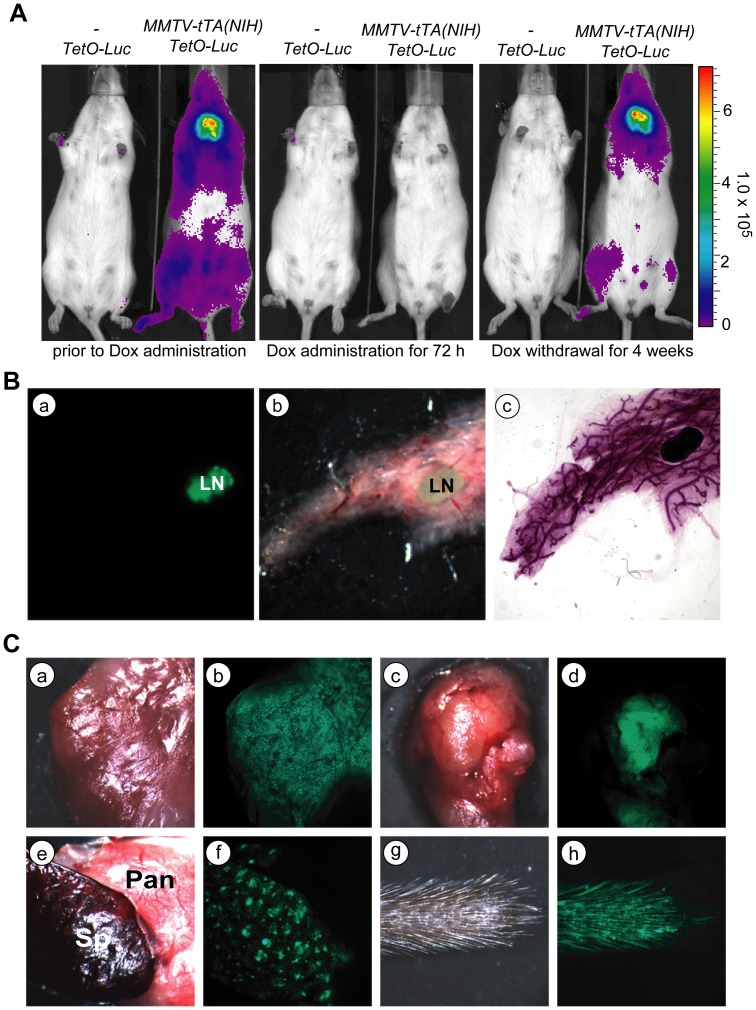
The MMTV-tTA(NIH) transgenic strain shows a strong tTA-mediated transactivation in many tissues. **A**. Bioluminescence imaging on a MMTV-tTA(NIH)/TetO-Luc double transgenic female and a TetO-Luc single transgenic littermate negative control. The three panels show the same animals prior to and 3 days after treatment with Dox as well as 33 days after Dox withdrawal. **B**. Fluorescence images of the GFP reporter expression in the mammary gland of a MMTV-tTA(NIH)/TetO-H2B-GFP double transgenic female. Panels a. and b. show GFP and bright-field images of an unfixed mammary gland. Panel c. shows the same gland after fixation and carmine red staining for visualizing mammary ducts. LN: lymph node. **C.** GFP expression in a variety of organs from a MMTV-tTA(NIH)/TetO-H2B-GFP double transgenic female; a, b: salivary gland; c, d: thymus; e, f: spleen (Sp) and pancreas (Pan); g, h: tail.

The initial analysis of the expression pattern of the tet-controlled transactivator in the MMTV-tTA transgenic strain generated by Hennighausen and colleagues [designated MMTV-tTA(NIH)] showed that the activation of the tTA in these mice was not restricted to the mammary gland [2]. Following the acquisition of this strain, which was carried in a mixed genetic background with C57/129 alleles at NIH, we transferred the MMTV-tTA transgene into an FVB background in our animal facility at UNMC with the intent to use this strain for mammary carcinogenesis studies. To investigate the temporal and spatial transactivation of TetO-driven responder genes mediated by this strain, we crossed the MMTV-tTA(NIH) transgenic line with TetO-Luciferase (TetO-Luc) reporter mice generated in our laboratory [5]. Using *in vivo* bioluminescence imaging, we confirmed that the MMTV-tTA(NIH)-mediated transactivation of the TetO-Luc transgene was widespread and not confined to the location of the ten mammary glands (Fig. 1A, left panel). Despite what appeared to be a ubiquitous expression of the MMTV-tTA, the transactivation of the tTA can be effectively controlled by doxycycline (Dox) in all target tissues. The administration of this ligand for 72 hours was sufficient to completely repress the expression of luciferase (Fig. 1A, middle panel), and the withdrawal of Dox led to a gradual reactivation of the TetO-Luc responder transgene in the salivary gland after two weeks and in the skin and mammary glands beginning at about four weeks (Fig. 1A, right panel).

To examine the expression of the MMTV-tTA in the mammary gland and other organs, we crossed the MMTV-tTA(NIH) line with TetO-H2B-GFP transgenic mice [8]. This reporter strain allowed for a more detailed analysis of the tTA-mediated transactivation in distinct tissues using a fluorescent stereo microscope (Fig. 1B, 1C). While the mammary gland lymph nodes exhibited a strong expression of GFP, the transactivation of the TetO-H2B-GFP transgene was very low and near the limit of detection in the mammary epithelium (Fig. 1B). Beside the lymph nodes in the mammary gland and other peripheral organs, we observed an exceptionally high expression of GFP in the salivary gland, thymus, spleen and skin (Fig. 1C). Table 1 summarizes the expression profile of the TetO-H2B-GFP reporter transactivated by the MMTV-tTA(NIH) transgene. Based on these observation using two different TetO-driven reporter transgenes, it is evident that the original MMTV-tTA(NIH) transgenic strain has limited applicability for the targeted expression of transgenes in the mammary epithelium in the FVB genetic background. In particular the activation of the MMTV-tTA in the skin makes it difficult to use this strain for *in vivo* imaging of the normal mammary gland and to monitor the development of mammary tumors. This was a primary motivation for the generation of new MMTV-tTA transgenic lines that may allow a more restricted expression of TetO-driven transgenes in the mammary epithelium.

**Table 1:** Tissue-specific expression profile of the TetO-H2B-GFP transgene under control of the original MMTV-tTA(NIH) strain and two novel MMTV-tTA transgenic lines (25754, 25755).

	Line NIH	Line 25754	Line 25755
Mammary epithelial cells	*	***	***
Salivary Gland (epithelial cells)	***	***	***
Thymus	***	No	No
Skin	***	No	No
Spleen	***	No	No
Pancreas	**	No	No
Liver	**	No	No
Kidney	*	No	No
Heart	*	No	No
Lung	*	No	No
Intestine	No	No	No
Skeletal muscle	No	No	No

*** high;

** moderate;

* low H2B-GFP expression efficiency; No; the tTA-mediated expression of H2B-GFP was undetectable.

### Generation of a new MMTV-tTA transgenic strain that permits a more defined temporally and spatially controlled expression of TetO-driven responder genes in the mammary epithelium

**Figure 2:**
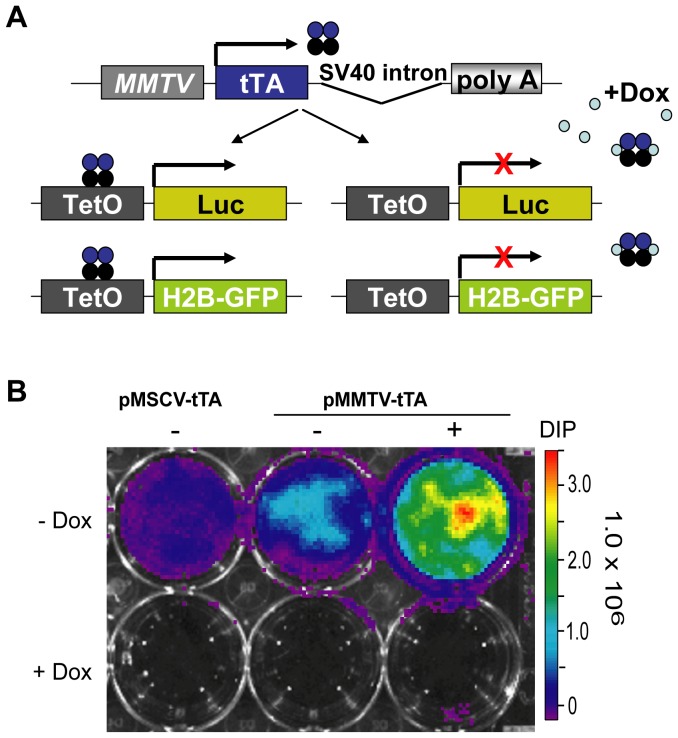
Cloning and functional analysis of a novel MMTV-tTA construct. **A**. Schematic outline of the new transgene and its applicability to control the expression of TetO-promoter driven reporter genes. **B**. Bioluminescence imaging of HC11 cells co-transfected with a TetO-luciferase plasmid and the MMTV-tTA construct or a pMSCV-tTA vector as a positive control. Transfected cells were cultured for 96 hours in the absence or presence of Dox (1 µg/ml). Note that the addition of lactogenic hormones (DIP: Dexamethasone, Insulin, and Prolactin) enhances the expression of the MMTV-tTA and luciferase.

To obtain a more restricted spatially controlled expression of TetO-inducible transgenes in mammary epithelial cells, we generated genetically engineered mice carrying the tet-controlled transactivator (tTA) under the regulation of the mouse mammary tumor virus long terminal repeat. While Hennighausen and colleagues had chosen a shorter MMTV LTR to generate the first MMTV-tTA strain, we inserted the tTA into the MMTV-SV40-BSSK vector (a kind gift from Dr. Muller, McGill University), which contains a longer LTR that includes a portion of the v-H-ras leader sequence (Fig. 2A). Based on previous reports [4], we expected that these regulatory elements mediate an enhanced expression of the tTA in the mammary gland. Following the cloning of the new MMTV-tTA construct, we verified its functionality by transfecting this construct along with a TetO-Luc reporter into mammary epithelial cells (HC11) that were cultured in the presence of hormones and growth factors that promote the proliferation and differentiation of these cells. The expression of luciferase was examined using bioluminescence imaging (Fig. 2B). A ubiquitously active pMSCV-tTA [7] vector was used as a positive control. A strong activation of the TetO-Luc reporter was observed when cells were maintained in EGF and insulin, but there was a further increase in the expression of luciferase when cells were treated with lactogenic hormones (DIP: dexamethasone, insulin, and prolactin). The activity of the reporter transgene was completely suppressed with Dox, which confirmed the correct functionality of the new MMTV-tTA transgenic construct.

**Figure 3:**
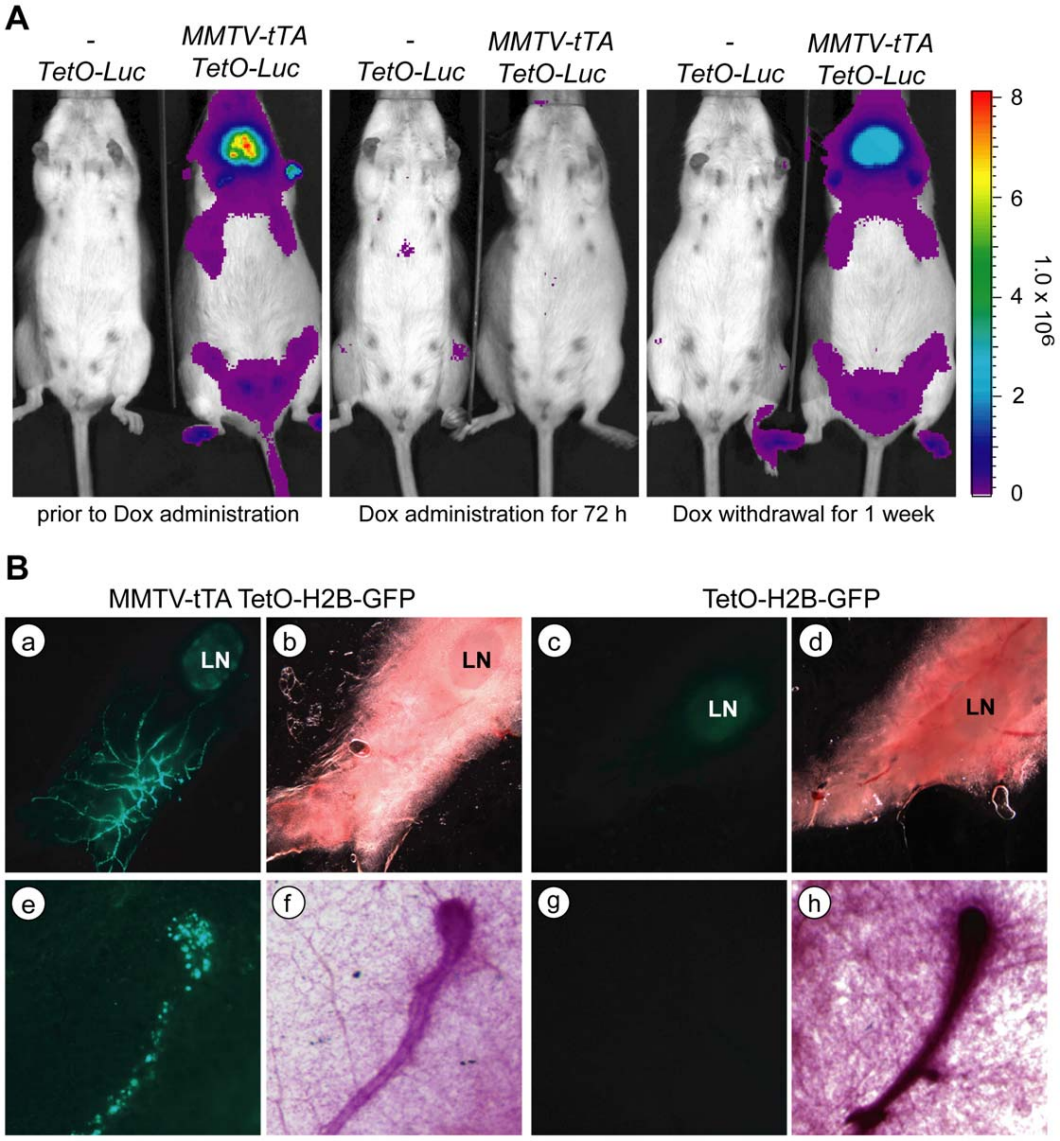
The novel MMTV-tTA transgenic strain allows for a defined and temporally controlled expression of TetO-driven responder genes in the mammary epithelium. **A**. Bioluminescence imaging of an MMTV-tTA/TetO-Luc double transgenic female and a TetO-Luc single transgenic littermate control (negative control) before and 3 days after administration of Dox as well as one week after withdrawal of Dox. **B**. GFP expression in a virgin mammary gland of an MMTV-tTA/TetO-H2B-GFP double transgenic female (a, b, e, f) and a single transgenic littermate control (c, d, g, h). Images were taken on viable tissues on a fluorescent stereoscope. The lower panels (e–h) show a higher magnification of the reporter gene expresses in a duct and terminal end bud. Note that there is evidence of GFP fluorescence in the lymph nodes of both the experimental animal and control suggesting that the reporter transgene exhibits a leaky expression in hematopoietic cells.

To generate transgenic mice, the 4.8 kb MMTV-tTA transgene was separated from the plasmid backbone with *Not*1 and injected into FVB zygotes. We obtained four transgenic founders of which two (#25754, and #25755) transmitted the transgene. To examine the tissue-specific expression pattern of the reporter transgene, we bred both founder lines with TetO-Luc reporter mice (Fig. 3A, left panel). Using *in vivo* bioluminescence imaging, we observed luciferase activity that was specific for the location of the thoracic and inguinal mammary glands, but there was also a high activation of the TetO-luc reporter in the salivary gland (Fig. 3A, left panel). The treatment of MMTV-tTA/TetO-Luc double transgenic mice with Dox resulted in a complete suppression of luciferase activity within 72 hours (Fig. 3A, middle panel), and the mice gradually regained expression of luciferase within only one week following withdrawal of Dox (Fig. 3A, right panel).

To determine the MMTV-tTA-mediated transactivation of responder genes in the mammary gland and other organs, we crossed both founder lines with the TetO-H2B-GFP reporter mice and analyzed unfixed tissues from the double transgenic offspring and their single transgenic littermate controls at four weeks of age under a fluorescent stereoscope (Fig. 3B). Expression of nuclear GFP was clearly visible in the mammary epithelial ducts of double transgenic females but not the controls. The GFP expression was particularly strong in large ducts descending from the nipple and in terminal end buds that are the proliferating distal regions of extending ducts. The weak expression of GFP that we observed in the mammary lymph nodes of double transgenic mice and their single transgenic controls was likely due to a leaky background activation of the TetO-H2B-GFP transgene as reported previously for hematopoietic stem cells [9]. The examination of GFP fluorescence in other tissues showed that, beside the mammary epithelium, the MMTV-tTA was only active in the salivary gland of both founder lines, but none of the other organs that were examined exhibited expression of H2B-GFP (Table 1). Hence, in comparison to the original MMTV-tTA(NIH) strain, both new lines permit a more defined expression of TetO-driven responder genes in the mammary epithelium in a temporally controlled manner. As anticipated from the experiments in hormone-treated mammary epithelial cells, a high expression of the MMTV-tTA was sustained in pregnant and lactating females (not shown).

### The novel MMTV-tTA strains exhibit high transactivation of TetO-driven responder transgenes in the perinatal mammary gland

**Figure 4:**
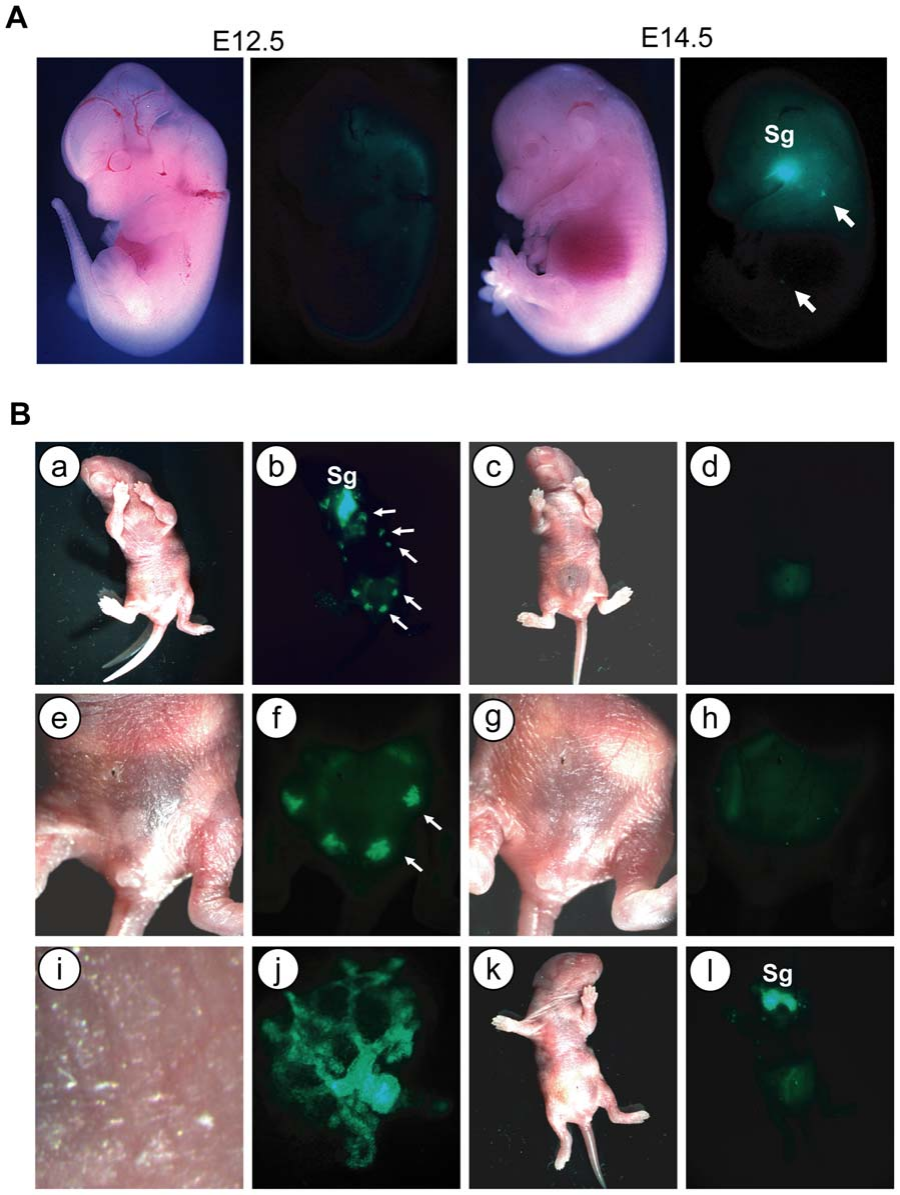
The new MMTV-tTA strain exhibits transgene activation in the perinatal mammary gland. **A**. GFP expression in MMTV-tTA/TetO-H2B-GFP double transgenic embryos on days E12.5 and E14.5. Bright-field (left) and GFP (right) images were taken on a fluorescent stereoscope equipped with a Spotflex camera. Sg, salivary gland; arrows indicate the location of the mammary placodes **B.** Expression of GFP in a newborn MMTV-tTA/TetO-H2B-GFP double transgenic female pup (a, b, e, f, i, j), a female TetO-H2B-GFP single transgenic negative control (c, d, g, h), as well as a male double transgenic littermate (k, l). The panels i. and j. show a higher magnification of the bright-field and GFP images of the nipple regions (arrows) in panels e. and f. Note that male pups lack mammary glands and, therefore, expression of GFP, yet exhibit strong activation of the reporter transgene in the salivary gland (Sg; panels k. and l.).

Since we observed a strong expression of the MMTV-tTA in the epithelium of mammary ducts near the nipple region but not in the adjacent skin, we were interested to know whether this transgene is already active in prepubescent females or even during perinatal mammary gland development. To address this question, we examined the expression of GFP in MMTV-tTA/TetO-H2B-GFP double transgenic embryos starting at day 10.5 of gestation when mammary gland anlagen/placodes begin to form. Using a fluorescent stereoscope, we observed a weak expression of GFP in the mammary buds and salivary glands at E13.5 (not shown), but GFP-labeled mammary gland anlagen were clearly visible from day E14.5 onward (Fig. 4A, arrows in the right panel). The expression of GFP was so strong in newborn pups that we were able to see their mammary glands without magnification using goggles with a GFP filter set and a modified flash light for excitation of the reporter protein (Fig. 4B. panels a and b). Upon examination under the fluorescent stereoscope, the entire mammary gland ductal tree became visible at higher magnification without the need of tissue dissection (Fig. 4B. panels a, b). With the exception of milk in the abdomen and a weak florescence in the fetal liver, control pups did not exhibit any notable expression of GFP (Fig. 4B. panels c, d and g, h). Interestingly, we only observed a high transactivation of the TetO-H2B-GFP reporter in the salivary glands of newborn double transgenic male pups (Fig. 4B. panels k, l). The areas of the rudimentary mammary glands in these animals were devoid of any expression of GFP. Using the special goggles, we were able to identify the double transgenic offspring right after birth as a result of the expression of GFP in the salivary gland, and we could easily identify the gender of the newborn offspring based on the mammary gland-specific expression of GFP in MMTV-tTA/TetO-H2B-GFP female pups. The reason for the gender-specific expression of the MMTV-tTA in the mammary gland relies on anatomical differences that are discussed in the last section of this article.

**Figure 5:**
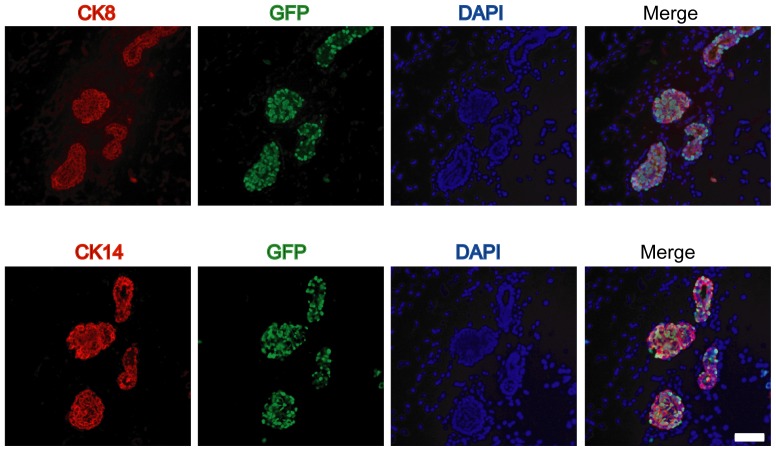
The MMTV-tTA is active in CK8/CK19 dual-positive bipotent mammary gland progenitors in newborn females. Immunofluorescence staining of GFP (green) as well as CK8 (red, upper panels) or CK14 (red, lower panels) in serial sections of a mammary gland from a newborn MMTV-tTA/TetO-H2B-GFP female. DAPI was used for counterstaining. The bar represents 50 µm.

At the perinatal stage, the mammary epithelium consists of quiescent immature epithelial progenitor linage that expresses both CK8 and CK14. After sexual maturity, these cells fully differentiate into luminal and basal epithelial lineages [10]. As demonstrated earlier, the MMTV-tTA construct in our novel transgenic lines was clearly active during perinatal mammary gland development. Using immunofluorescence staining on histological sections of the mammary gland of newborn females, we subsequently confirmed that the MMTV-tTA transgene is expressed in mammary epithelial progenitors that express CK8 and CK14 (Fig. 5).

### The MMTV-tTA-mediated expression of Cre recombinase permits the deletion or activation of genes in luminal and basal epithelial cells

**Figure 6:**
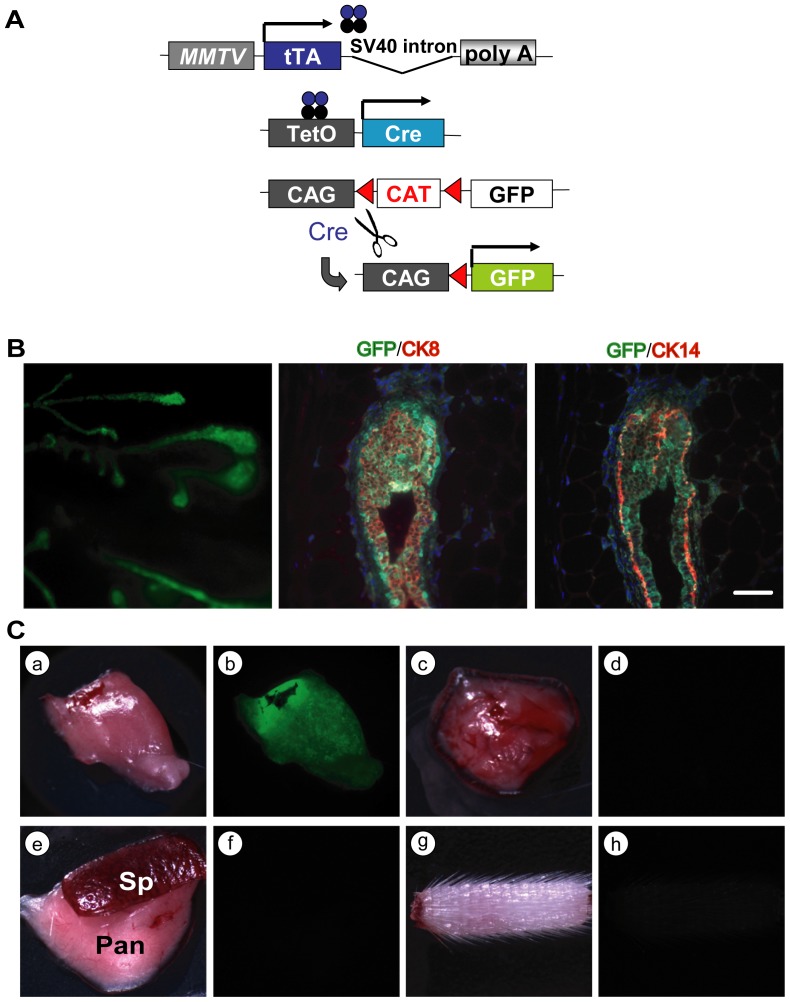
Genetic labeling of luminal and basal mammary epithelial cells in adult females. **A**. Schematic outline of the experimental approach to permanently activate GFP under regulation of the constitutively active CAG promoter in cells expressing Cre recombinase under control of the MMTV-tTA. **B**. CAG-GFP expression in mammary ducts of an MMTV-tTA/TetO-Cre/CAG-GFP triple transgenic female. The left image was taken under a fluorescent stereoscope. The middle and right panels show a serially sectioned terminal end bud that was stained against GFP (green) as well as CK8 or CK14 (red). The bar represents 50 µm. **C**. GFP expression (or lack thereof) in the variety of organs from a triple transgenic female; a, b: salivary gland; c, d: thymus; e, f: spleen (Sp) and pancreas (Pan); g, h: tail.

Since the MMTV-tTA is expressed in mammary gland progenitors during embryonic development and in newborn females, we reasoned that a transactivation of TetO-driven transgenes expressing Cre recombinase should lead to a permanent labeling of luminal and basal mammary epithelial lineages in adult mice. To experimentally address this question, we crossed double transgenic mice that carry a TetO-Cre transgene in addition to the MMTV-tTA with a Cre/lox reporter strain that expresses cytoplasmic GFP upon Cre-mediated recombination (CAG-lox-CAT-lox-GFP or short CAG-GFP [11]). A schematic outline of the permanent labeling of MMTV-tTA-expressing cells is shown in Fig. 6A. Using this system, the tTA-expressing progenitors and their derived descendants are being labeled with GFP at the perinatal stage. The resulting expression of the fluorescent marker under the constitutive CAG promoter was strong in the entire mammary epithelium of adult triple transgenic females (Fig. 6B, left panel). As anticipated, the fluorescent co-staining of GFP with CK8 or CK14 confirmed that the MMTV-tTA-mediated expression of the TetO-Cre was targeted to bipotent progenitors and, possibly, also their more differentiated descendants that maintain expression of the MMTV-tTA in adult females (Fig. 6B, middle and right panel). Overall, this strategy to genetically label MMTV-tTA-expressing cells on the basis of Cre recombinase appears to be much more sensitive than the tTA-mediated transactivation of the TetO-H2B-GFP reporter. One reason for this phenomenon is that the levels of GFP in cells with either strong or weak MMTV-tTA expression are equally high since little expression of Cre is sufficient to constitutively activate the CAG-GFP reporter. Hence, this system is very sensitive and would also detect low levels of MMTV-tTA activity in other organs. Despite the increase in sensitivity, we did not observe any GFP fluorescence in tissues other than the mammary and salivary glands (Fig. 6C). Collectively, these observations suggest that the novel MMTV-tTA strains facilitate a targeted transactivation of responder genes in these two secretory organs. In addition, these lines are expected to be useful tools for a TetO-Cre-mediated deletion of conditional knockout alleles.

### Expression of the MMTV-tTA is sustained during ErbB2-induced mammary tumorigenesis

**Figure 7:**
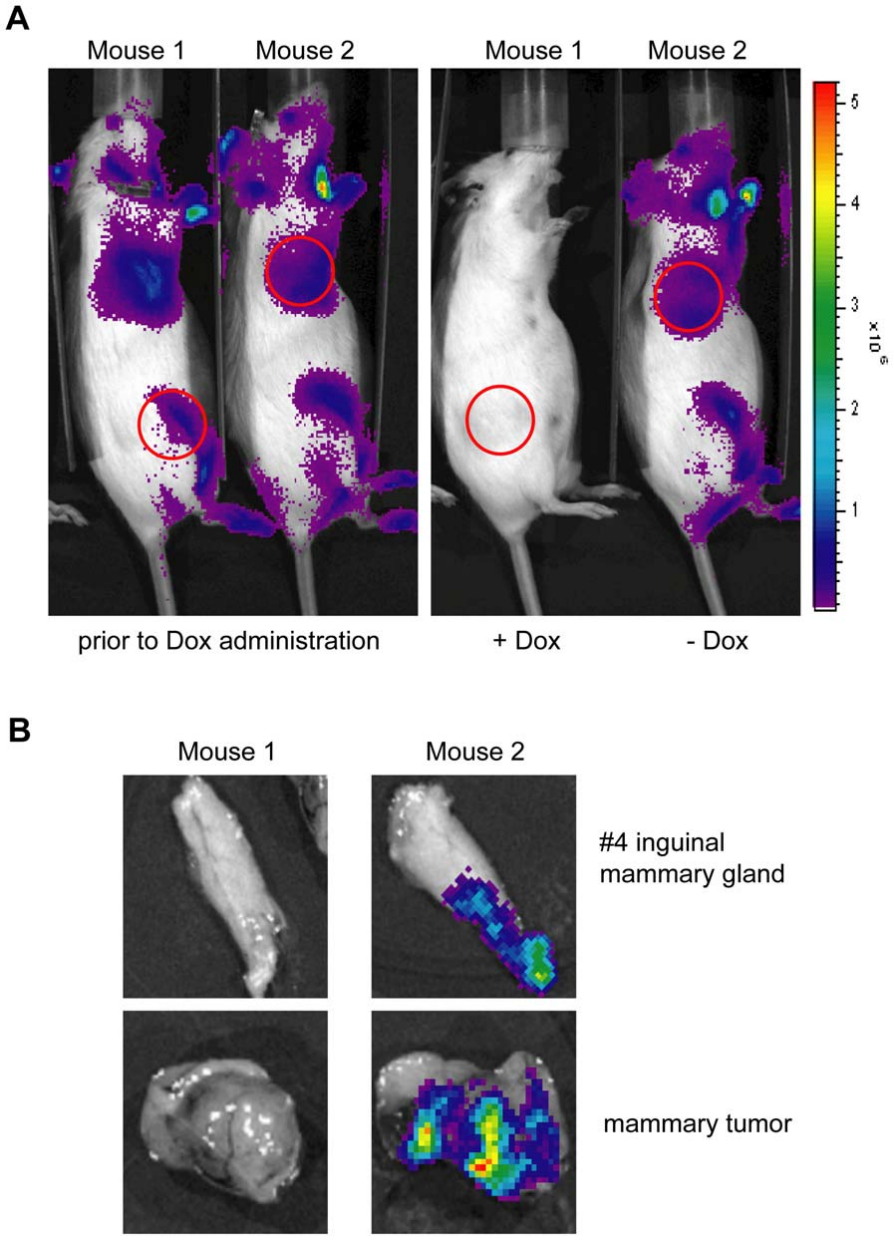
ErbB2-induced mammary tumors continue to express the MMTV-tTA transgene. **A**. Bioluminescence imaging on two MMTV-neu/MMTV-tTA/TetO-Luc triple transgenic females that carry mammary tumors (red circles). The left panel shows both mice prior to treatment with Dox. Only female #26645 received Dox in the drinking water for 3 days, and the right panel shows the expression of luciferase (or lack thereof) after Dox treatment. **B**. Bioluminescence images on dissected mammary glands and tumors of both mice shown in panel A, i.e. 3 days of Dox treatment (#26645) and untreated control (#26656).

Based on the expression profile of the novel MMTV-tTA strains, it is evident that these mice could be used to target a constitutive expression of oncogenes to the mammary epithelium throughout normal mammogenesis without administration of Dox. If these oncogenes are equally required for tumor initiation, growth, and maintenance, it can be expected that neoplastic cells are selected for expression of the MMTV-tTA transgene. On the other hand, it might be desired to target transgenes to neoplastic cells that are not directly involved in the process of neoplastic transformation. Such experiments might include the use of phenotypically neutral reporter genes to monitor neoplastic growth and metastasis using *in vivo* imaging. In this case, it is essential that the expression of the MMTV-tTA exhibits a low rate of mosaicism, and the activity of the tTA must be maintained in neoplastic cells. Based on the low expression levels of the original MMTV-tTA(NIH) in the mammary gland, we were unable to detect any expression of the TetO-Luc transgene in most MMTV-neu-induced mammary tumors (Zhang and Wagner, unpublished). To test whether the expression of the tTA is being maintained in the novel MMTV-tTA strains following the onset of mammary carcinogenesis, we crossed MMTV-tTA/TetO-Luc double transgenics into the MMTV-neu strain. As reported previously [12]–[14], the majority of triple transgenic females that overexpressed wildtype ErbB2 developed CK8-positive, luminal-type mammary tumors after a medium latency of more than 6 months in the FVB background (data not shown). Using bioluminescence *in vivo* imaging on these triple transgenic females, we were able to detect expression of the TetO-Luc transgene in palpable mammary tumors (Fig. 7A, left panel), which is indicative of a sustained expression of the MMTV-tTA during mammary carcinogenesis. Using treatment of tumor-bearing mice with Dox, we confirmed that the MMTV-tTA-mediated transactivation of the luciferase reporter gene could be effectively suppressed in growing mammary tumors (Fig. 7A, right panel and Fig. 7B). Collectively, this line of investigation clearly shows that the novel MMTV-tTA strains are useful to target the expression of transgenes into developing mammary tumors. Such an approach might be valuable to assess the role of particular genes for tumor metabolism or metastatic dissemination.

## Discussion

The ability to spatially and temporally regulate transgenes in mammary gland *in vivo* is useful for studying the function of proteins at a variety of physiological, developmental, and disease-related processes. In order to develop the necessary tools for such *in vivo* experiments, we generated a novel transgenic strain that expresses the tetracycline-controlled transactivator (tTA, Tet-OFF) under the regulation of an MMTV-LTR that carries a portion of the v-H-ras leader sequence [4]. The analysis of various reporters under control of the MMTV-tTA transgene clearly demonstrated that, in contrast to a similar strain generated nearly two decades ago [2], the new transgenic lines permit a more restricted expression of TetO-driven responder transgenes in the mammary gland in a ligand-controlled manner. These new lines complement the currently used MMTV-rtTA strain generated by Gunther et al. [3], which exhibit a strong activity of the reverse transactivator (rtTA, Tet-ON) in mammary epithelial cells. MMTV-rtTA mice have been utilized to target the expression of potent oncogenes, such as ErbB2 [15], Wnt1 [16], c-Myc [17], and K-Ras [18] to the mammary gland in a temporally controlled fashion. These models typically developed mammary tumors after a short or medium latency while the animals were treated with Dox. There are, however, a number of growth factor receptors, transcription factors, and cell cycle regulatory proteins that are known to contribute to breast cancer initiation in humans and mice, but their oncogenicity *in vivo* is generally low. For example, expression of prolactin and its effector Stat5 in the mammary gland of transgenic mice lead to carcinogenesis after latencies of more than 12 to 15 months [19], [20]. To overexpress these proteins in a temporally controlled manner under control of the MMTV-rtTA, it would require a constant administration of Dox to these animals, which is also costly since the drug has to be frequently replaced when administered in the drinking water. We have reported previously that treatment of animals with this drug over a long time can lead to poor health, obesity, and diabetes due to the high energy supplements in the food or water that are intended to mask the bitter taste of Dox [7]. Our novel MMTV-tTA might, therefore, be well suited for long-term studies that require a constitutive expression of genes over a prolonged period without the need for the administration of Dox. The subsequent downregulation of these genes to test their biological relevance during development or their therapeutic efficacy in cancer would require a comparatively short treatment with the drug, which mimics more closely a therapeutic intervention where oncogenic signaling pathways are being repressed for a limited time.

One of the important characteristics of the new MMTV-tTA strain is its ability to transactivate TetO-driven responder genes at very early stages of mammary gland development. Unlike the MMTV-tTA(NIH) strain, which exhibits a widespread expression in the embryonic ectoderm (not shown) and in the skin of adult animals, the new transgenic lines show expression of the tTA specifically in mammary gland anlagen starting at day 13 of gestation. Hence, the new MMTV-tTA transgenics might be uniquely applicable to study genes that regulate the earliest stages of mammary gland development in the embryo. The expression of the MMTV-tTA was first observed in both male and female embryos at a time when mice begin to develop a sexual dimorphism in the embryonic gland. In most mouse strains, males do not have nipples due to the action of androgens starting at day E13, which elicits regression of the embryonic mammary gland by E16 [21], [22]. This explains why we were able to easily distinguish the gender of newborn MMTV-tTA/TetO-H2B-GFP pups by simply examining the fluorescence of GFP in the inguinal and thoracic regions of the ten mammary glands.

Transplantation studies using mammary gland anlagen have demonstrated that cells of the embryonic gland are multipotent [22]. While it has been suggested for more than 30 years that multi-potent stem cells may arise at E13 to E17 [23], Spikes et al. recently provided experimental evidence that the number of fetal mammary stem cells peaks in late embryogenesis [24]. Mammary progenitor cells continue to express both luminal and basal markers (CK8 and CK14, respectively) prior to puberty [10]. Our observations clearly show that the MMTV-tTA is active in multipotent mammary progenitors that are positive for CK8 and CK14. This provides new opportunities to utilize the MMTV-tTA strain to genetically label or isolate embryonic mammary gland stem cells and to perform lineage tracing experiments using the TetO-Cre responder transgene in combination with a Cre/loxP reporter transgene, such as the CAG-GFP transgenic strain. Since it has been observed that the activity of the MMTV promoter increases in response to the levels of lactogenic hormones, it is frequently assumed that these regulatory elements are predominantly active in differentiated luminal epithelial cells. It is, however, evident that the MMTV provirus is capable of infecting mammary stem cells, which has been used to trace single mammary progenitors [25]. In support of this notion, the longitudinal analysis of GFP expression in MMTV-tTA/TetO-H2B-GFP double transgenic embryos and adult females provides supporting evidence that the MMTV-LTR is, indeed, expressed in multipotent mammary epithelial progenitors and not restricted to their differentiated descendents. Therefore, the new MMTV-tTA transgenic strain might be useful to target the expression of exogenous proteins to multipotent progenitors to study their importance for pre- and postnatal mammary gland development.

## Materials and Methods

### Cloning and verification of functionality of the MMTV-tTA transgene in mouse mammary epithelial cells

The tTA coding sequence was released as an *Eco*RI/*Bam*HI blunted fragment from pMSCV-tTA-IRES-GFP [7]. The tTA sequence was directly inserted into the blunted *Eco*RI site of MMTV-SV40-BSSK vector (provided by Dr. Muller, McGill Univ.) that has been modified at the *Sal*I site as *Sal*I-*Spe*I-*Not*I-*Spe*I-*Sal*I sequence. The functionality of the MMTV-tTA construct was confirmed by transfecting it along with a TetO-Luciferase reporter construct into mouse mammary epithelial cell lines, HC11, using Fugene (Roche) according to the manufacturer's protocol. The cells were maintained in DMEM:F12 supplemented with 10% fetal bovine serum, and the lactogenic hormones, DIP (Dexamethasone; 5 µM, Insulin; 1 µg/ml, and Prolactin; 1 µg/ml). Those cells were also treated with Doxycycline (1 µg/ml) to suppress the expression of the reporter transgene. After 72 hrs transfection, the cells were administrated with luciferin (150 µg/ml) and the expression of luciferase was quantified using bioluminescence imaging (IVIS200, Caliper Life Science, Alameda, CA).

### Generation of MMTV-tTA transgenic mice and genotyping protocols

The MMTV-tTA insert was released from the vector as a *Not*I-*Not*I fragment 4.8 kb in size. This DNA fragment was injected into FVB zygotes at a concentration of 1–10 ng/µl. The pronuclear injection was performed at the UNMC Mouse Genome Engineering Core Facility. For this study, we examined two MMTV-tTA transgenic founder lines, # 25754 and 25755 [Tg(MMTV-tTA)25754Kuw, Tg(MMTV-tTA)25755Kuw], that exhibited a virtually identical expression pattern. The MMTV-tTA transgene was detected by PCR using primers corresponding to the MMTV LTR and the tTA coding sequence (2127; 5′- AGT GAT AGA GCT CTT GCC TAG C-3′ and 599; 5′- GCC AAT ACA GTG TAG GCT GC-3′). Alternatively, we used primer sets detecting the 3 prime end of the tTA sequence and the SV40 intron/polyA sequence (421; 5′- CGC TAG ACG ATT TCG ATC TGG-3′ and 2128; 5′- CTC CCA TTC ATC AGT TCC ATA GG-3′). The resulting PCR products were 364 bp and 370 bp, respectively. TetO-Luciferase (TetO-Luc) transgenic mice were genotyped as described previously [5]. TetO-H2B-GFP [8] and MMTV-neu [12] transgenic mice were obtained from the Jackson Laboratory. The CAG-lox-CAT-lox-GFP (short CAG-GFP) reporter strain [11] was kindly provided by Dr. Miyazaki (Osaka University Medical School). All mice were maintained in an FVB genetic background. This study was carried out in strict accordance with the recommendations in the Guide for the Care and Use of Laboratory Animals of the National Institutes of Health. The protocol was approved by the Institutional Animal Care and Use Committee of the University of Nebraska Medical Center (IACUC#: 03-104-01).

### In vivo imaging of luciferase and GFP expression

The expression and activity of the luciferase reporter gene was monitored using *in vivo* bioluminescence imaging machine (IVIS200) as described previously [5], [7]. To determine the H2B-GFP reporter gene activation in embryonic tissues and other organs, including the mammary gland, tissues were isolated from MMTV-tTA/TetO-H2B-GFP double transgenic mice, and reporter gene expression was assessed under a fluorescent stereo microscope (Discover.V8, Carl Zeiss) equipped with a SPOT FLEX camera (Diagnostic Instruments, Sterling Heights, MI).

### Whole-mount and immunofluorescent staining

Whole mounts were prepared and stained with carmine alum as described previously [26]. The mammary glands were fixed with buffered formalin and sectioned. Details about the immunofluorescent staining of CK8 and CK14 as well as GFP were described previously [5], [14], [27]. We used the following primary antibodies: CK8 (Troma II, 1∶250 dilution) from the Iowa Hybridoma Bank), CK14 (1∶1,000 dilution) from Convance, and GFP (GFP-1020, 1∶500) from Aves Labs. Images of histological slides were taken on a Zeiss AxioImager microscope (Carl Zeiss) equipped with a SPOT FLEX camera.
